# Using physical elicitors to enhance pigment production in *Streptomyces* sp. VITGV38, and bioactivity and molecular docking studies of the extracted pigment-related compounds

**DOI:** 10.5114/bta/215700

**Published:** 2026-03-13

**Authors:** Elumalai Soniyagandhi, John Godwin Christopher

**Affiliations:** Department of Biomedical Sciences, School of BioSciences and Technology, Vellore Institute of Technology, Vellore, India

**Keywords:** ADMET, antimicrobial activity, antioxidant activity, docking, GC-MS, pigments

## Abstract

**Background:**

Actinomycetes are soil-dwelling microorganisms known for the production of natural pigments, which serve as alternatives to synthetic dyes. In this study, the effects of natural light and total dark conditions on pigment production in *Streptomyces* sp. VITGV38 (MCC 4869) were examined, and the extracted pigment-related compounds were subjected to pharmacokinetic analysis, molecular docking studies, and evaluation of antioxidant and antibacterial activities.

**Materials and methods:**

The *Streptomyces* strain was cultured under complete darkness and natural (light/dark) conditions to compare pigment yield and bioactivity. Antioxidant activity of the crude pigment extract was determined by the DPPH free radical scavenging assay. Antibacterial activity of the pigment extract was tested against *Escherichia coli, Staphylococcus aureus, Bacillus subtilis*, and *Pseudomonas aeruginosa*. Gas chromatography-mass spectrometry (GC-MS) was conducted to identify individual compounds of the pigment extract. Molecular docking of the pigment-related compounds was performed using AutoDock Vina, and their pharmacokinetic properties were predicted using SwissADME.

**Results:**

After 21 days of culturing the strain under light conditions, the pigment extract at 100 µg/ml concentration showed the maximum antioxidant activity of 83.73% with an IC_50_ value of 0.143 µg/ml, indicating strong antioxidant capacity. The antibacterial activity of the pigment extract was higher when the strain was cultured dark conditions. Gas chromatography-mass spectrometry analysis identified seven major compounds in the pigment extract: n-hexadecanoic acid, tetracosane, pentadecanoic acid, tetradecanoic acid, indole, benzo(h)quinoline-2,4-dimethyl, and 2-piperidinone. Molecular docking analysis revealed strong interactions of the pigment-related compounds with potential protein targets. SwissADME analysis showed that the pigment-related compounds have favorable drug-like properties.

**Conclusions:**

*Streptomyces* sp. VITGV38 is a promising source of bioactive pigments with potent antioxidant and antibacterial activities. While culturing the strain under natural light conditions enhanced the antioxidant capacity and yield of the pigment, culturing under dark conditions enhanced the antibacterial activity of the pigment. These findings highlight the multifaceted potential of Streptomyces sp. VITGV38 for developing natural antimicrobial agents.

## Introduction

Actinomycetes are Gram-positive, filamentous bacteria with a relatively high G+C content in their DNA. The genus *Streptomyces*, a member of the *Actinomycetes* group, produces several beneficial secondary metabolites (Mesrian et al. 2021). *Streptomyces* are frequently used in biotechnological applications to synthesize secondary metabolites such as antibiotics, pigments, and enzymes. They also possess anti-inflammatory, antioxidant, antimicrobial, and anticancer properties (Polapally et al. 2022). Certain actinomycetes form colored colonies during their growth through the production of pigments in various shades, including yellow, orange, red, blue, violet, and brown (Udhyakumar et al. 2017). These pigments serve as excellent natural alternatives to synthetic dyes. Because of their ability to produce a wide range of pigments, actinomycetes are a promising source of edible colorants. Pigments play critical roles in various industries, particularly in the food industry, where they function as additives, color intensifiers, and antioxidants (Malik et al. 2012). Pigment-based colors are widely used in cosmetics, artwork, plastics, textiles, food coloring, and pharmaceuticals (Singh Parmar et al. 2016).

Fermentation technology facilitates the large-scale, cost-effective production of microbial pigments. Compared to synthetic pigments, these natural pigments are biodegradable, nontoxic, and highly stable (Kamarudheen et al. 2019) Some notable antimicrobial pigments produced by *Streptomyces* species include undecylprodigiosin (red) from *Streptomyces* sp. BSE6.1 (Ramesh et al. 2021), carotenoid (yellow) from *Streptomyces* strain AQBMM35 (Dharmaraj, 2009), actinorhodin (blue) from *S. coelicolor* (Palanichamy et al. 2011), and metacycloprodigiosin (red) from *S. spectabilis* (Meng-xi et al. 2021)

Recent studies have explored elicitors as a novel approach to enhance microbial metabolite production. Elicitors activate specific transcription factors and upregulate unique genes, thereby initiating metabolic pathways. The introduction of elicitors in the intra/extracellular environment of microorganisms induces stress, causing them to increase secondary metabolite production. Elicitors can be classified as biotic (derived from living organisms) or abiotic (derived from nonliving sources) (Bhaskar et al. 2022). Reactive oxygen species (ROS) are generated during normal cellular metabolism. However, excessive levels of ROS can lead to oxidative stress, which is associated with various diseases, including cancer, rheumatoid arthritis, cataract, atherosclerosis, and ischemia-reperfusion injury (Lee et al. 2014). Recent research has increasingly focused on the use of microbial- and plant-derived natural antioxidants as safe therapeutic agents (Radhakrishnan et al. 2016). Molecular docking simulations can be used to validate the binding interactions of enzymes with a wide range of ligands. These simulations employ various binding models to determine the active regions of enzymes. Additionally, Absorption, Distribution, Metabolism, and Excretion (ADME) properties of secondary metabolites can be assessed using specialized computational techniques. Potential therapeutic candidates showing an optimal crossover efficiency of 50% undergo pharmacokinetic evaluations and ADME/toxicity tests before advancing to the drug development stage. *In vitro* ADME/toxicity studies are a crucial step in drug development, although they are time-consuming and expensive (Kumari et al. 2023).

Light, as a physical elicitor, can influence secondary metabolite production (Anasori and Asghari 2009). Solvents with different polarities, such as ethyl acetate, chloroform, methanol, and ethanol, are commonly used to extract pigments (Srinivasan et al. 2017). Given this background, the present study investigated the effects of natural light and total dark conditions as physical elicitors on the antimicrobial activity and pharmacokinetic properties of the pigment extracted from *Streptomyces* sp. VITGV38. Additionally, antioxidant assay, ADME/toxicity analysis, and molecular docking studies were conducted to elucidate the antioxidant activity, toxicity, and drug-likeness of the extracted pigment, respectively.

## Materials and methods

### Isolation of Streptomyces sp. VITGV38 (MCC 4869)

Endophytic *Streptomyces* strain VITGV38 (MCC 4869) was isolated from a tomato plant and grown on ISP2 agar. To achieve log phase, the strain was cultured for 10 days at 37°C. Next, 300 ml of broth (pH 7.0) was added to a 500 ml conical flask and inoculated with a 10-day-old culture. The flask was incubated on an orbital shaker at 130 rpm for 30 days at 30°C (Veilumuthu and Godwin 2022).

### Morphological characteristics

*Streptomyces* sp. VITGV38 culture from ISP2 agar was transferred to starch casein agar (SCA) plates and cultivated for 10 days to promote pigment formation. After examining the morphological features of the colony under a magnifying glass, phase-contrast microscopy was used to evaluate the color of the aerial mycelium, colony shape, pigmentation, and mycelial structure.

### Scanning electron microscopy

The colonies of *Streptomyces* sp. VITGV38 were observed for aerial mycelia, substrate color, and growth intensity. Scanning electron microscopy (SEM) (Evo 18, Carl Zeiss, Japan) was used to examine spore morphology. To obtain fine structural details, mycelial spores were meticulously mounted onto a carbon stub, coated for conductivity, and subsequently imaged.

### Natural (light and dark) and total dark conditions

One set of experiments was conducted under natural light conditions (day/night (14 : 10)) with three 500 ml flasks each containing 300 ml of *Streptomyces*-inoculated culture media for different durations (7, 14, and 21 days), In the second set of experiments, the *Streptomyces* strain was cultured in another 3 flasks under the total darkness condition achieved by covering the flask with a black plastic paper. An optimum pH of 7.0 was maintained based on the previous experiment. The flasks were placed on a shaker at 130 rpm and incubated at 30°C.

### Pigment extraction

Following the culturing period, the culture medium was centrifuged at 5000 rpm for 15 min at 4°C to extract secondary metabolites; the supernatant was then filtered through a Whatman filter paper to obtain cell-free culture filtrate. Pigments from 7-, 14-, and 21-day-old culture were extracted by vigorously shaking the culture filtrate with ethyl acetate (1 : 1, v/v) at 150 rpm overnight. The organic phase (ethyl acetate extract) was collected after separating the organic and aqueous phases by using a separating funnel. A rotary evaporator (Eyela N-1000, Japan) was then used to concentrate the extract, which yielded the crude pigment extract. Individual compounds of the extract were identified by gas chromatography-mass spectrometry (GC-MS).

### GC-MS

The GC-MS analysis was conducted using Trace GC Ultra coupled with the ISQ Single Quadrupole MS (Thermo Scientific, Waltham, MA), with a TG-5MS fused silica capillary column (30 m × 0.25 mm, 0.1 mm film thickness). Metabolites were identified using an electron ionization system with an ionization energy of 70 eV. The inert gas helium was used as a carrier gas at the flow rate of 1 ml/min. The MS transfer line and injector were maintained at 280°C. The temperature was gradually increased from 50°C in 2 min to 150°C in 7 min and then from 270°C to 310°C in 3.5 min.

### Antimicrobial activity assay

The antimicrobial activity was determined using the agar well diffusion method on Mueller-Hinton agar. Secondary metabolites from three culture batches (7^th^ day, 14^th^ day, and 21^st^ day cultures) cultivated under natural (light/dark) and total dark conditions were evaluated for antibacterial activity against four test pathogens (*Escherichia coli, Staphylococcus aureus, Bacillus subtilis*, and *Pseudomonas aeruginosa*). Cultures of the test pathogen strains were spread onto the agar plates, and a 6-mm borer was used to create wells in the solidified agar. Crude pigment extracts were added to each well, with ethyl acetate and tetracycline as negative and positive controls, respectively. Antibacterial activity was determined by measuring the inhibition zones with a zone-measuring scale.

### Antioxidant activity

The pigment extracts were tested for their ability to scavenge DPPH (2,2-diphenyl-1-picrylhydrazyl) radicals. A DPPH solution (0.002% in methanol) was prepared for the assay, and ascorbic acid served as the reference standard. Each crude extract sample was tested at a concentration of 0.1 mg/ml. Briefly, 2 ml of crude extract samples (from both natural and dark conditions) and 2 ml of DPPH solution were mixed in different test tubes. After vigorous shaking, the tubes were incubated for 30 min in the dark. A UV-Vis spectrophotometer was used to detect absorbance at 517 nm, with methanol as the blank. The percentage of radical scavenging activity was calculated, and IC_50_ values were determined based on the reduction in absorbance values.

### Pharmacokinetic studies

The ADME web server was used to study the pharmacokinetic characteristics of the identified compounds to determine their potential as drug candidates. The ADME profile reflects the pharmacological activity, bioavailability, and tissue exposure characteristics of a compound. Lipinski’s rule of five, a crucial criterion in drug development, was used to evaluate the drug-likeness of each compound. Nine different compounds were examined, and the compounds that met the drug-likeness requirements were chosen for testing as potential therapeutic agents by using molecular docking analysis.

### Molecular docking analysis

Molecular docking was performed using AutoDock Vina integrated with PyRx in accordance with the protocol established by Ul Hassan *et al*. (2021). Based on earlier research and the co-crystallized ligand in the recovered protein structures, the binding site residues of the target proteins were determined. The binding pocket coordinates were established using BIOVIA Discovery Studio. Selected secondary metabolites of *Streptomyces* sp. VITGV38, such as benzo(h)quinoline-2,4-dimethyl and indole, were obtained in SDF format from PubChem, translated to PDB format using PyRx, and further prepared for docking in PDBQT format using AutoDock Tools by adding Gasteiger charges and polar hydrogen elements. To minimize energy use, 3D structures were generated using OpenBabel. 5IMJ (*E. coli*), 3G75 (*S. aureus),* 3E7X (*B. subtilis*), and 4QMK (*P. fluorescens*) were the target proteins downloaded from the Protein Data Bank (PDB). By eliminating water molecules and preexisting ligands, these target proteins were preprocessed in BIOVIA Discovery Studio. Next, Kollmann charge optimization was performed, and polar hydrogen atoms were added using AutoDock Tools. Binding affinity was assessed using binding energy scores and interaction types. Docking was conducted using AutoDock Vina coupled with PyRx 0.8. BIOVIA Discovery Studio was used to view docked structures and identify functional annotations and binding pockets. To validate the docking protocol, redocking was performed by reinstating the original co-crystallized ligand into the active site of each target protein. The RMSD (Root Mean Square Deviation) values were calculated by comparing the redocked ligand to the original co-crystallized pose. The grid box and docking parameters were carefully defined to accurately include the active site residues, ensuring reliable ligand placement. The RMSD values were obtained using Pose Viewer in PyRx.

## Results

### Isolation and culturing of Streptomyces sp. VITGV38 (MCC 4869)

The *Streptomyces* strain was obtained from the VIT University Microbiology Laboratory and was originally isolated from tomato plants. The strain was cultured on an ISP2 agar medium and incubated at 30°C for 10 days. High pigment production was observed on SCA. The isolate was then subcultured and maintained for further experiments.

### Morphological characteristics

**Figure 1:**
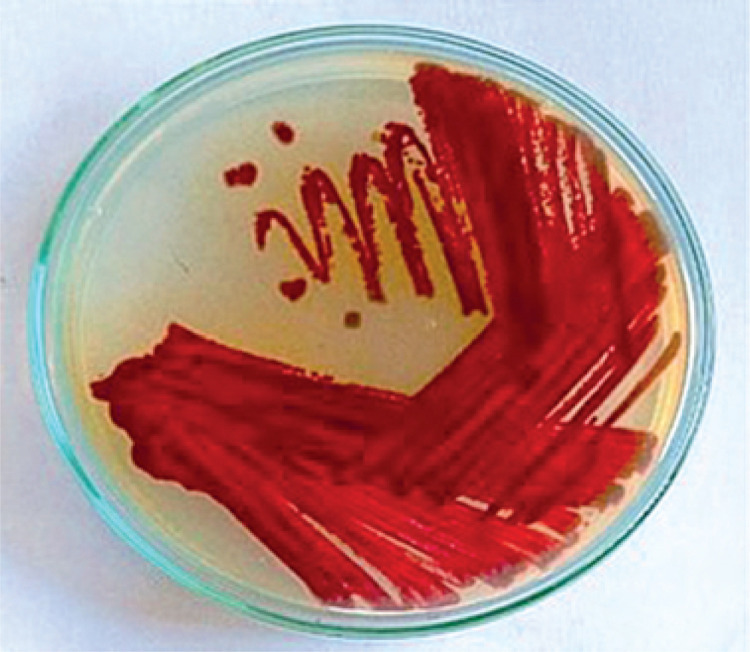
Shows the pigment produced by Strepto myces sp. VITGV38 on Starch Casein Agar media

The *Streptomyces* strain was morphologically characterized on SCA medium. The strain produced spores on the 3^rd^ day of the culture, and a pink pigment was observed in the culture medium. By the 7^th^ day, the color had transitioned from pink to dark brown. The surface of the colonies was smooth, with grey aerial mycelia and slightly pinkish substrate mycelia. Figure 1 shows the color transition and surface morphology, supporting the identification of melanin-like pigment production.

### SEM

**Figure 2:**
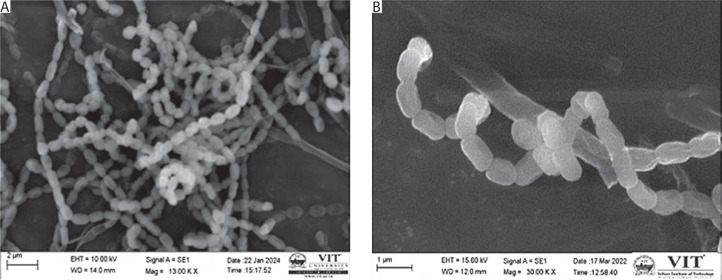
Scanning electron microscopy morphology of Streptomyces sp. VITGV38

SEM imaging was used to examine the spore morphology and surface structure of *Streptomyces* sp. VITGV38. The isolate showed tightly coiled hyphae with bilobed branches and a smooth, greyish, powdery spore surface. The spores were convex and arranged in an extended, compressed spiral chain, consisting of approximately 15 to 20 spores per chain. The spores were cylindrical in shape, with the average width and length of 0.5 and 0.8 µm, respectively. Figure 2 illustrates the typical morphology of the *Streptomyces* strain.

### Pigment extraction

Pigment production was observed from the 7^th^ day in liquid culture. By the 21^st^ day, cell masses were formed. Secondary metabolites from the *Streptomyces* strain were extracted at three time points (7, 14, and 21 days) by using ethyl acetate (1 : 1) as the solvent. The organic phase was separated from the aqueous phase using a separating funnel and concentrated using a rotary evaporator. The crude extract was subsequently analyzed by GC-MS.

### GC-MS

**Figure 3:**
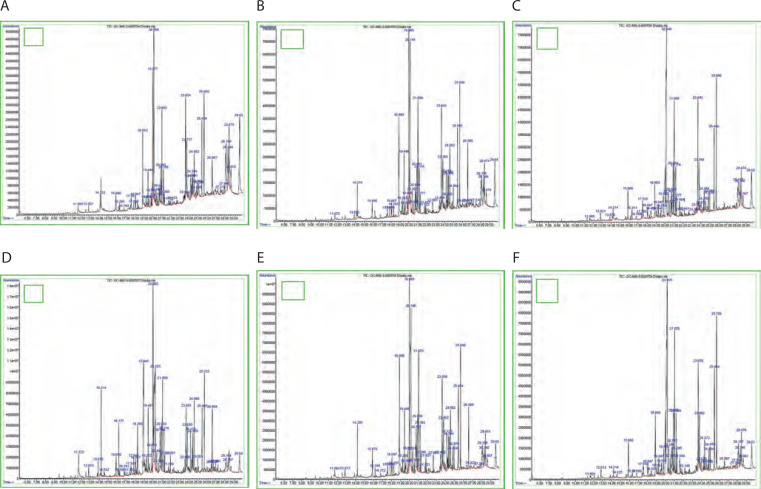
Shows that based on GC-MS analysis, the chromatogram of the bioactive compounds on ethyl acetate crude extract of Streptomyces sp. VITGV38. (**A**) Light 7 days, (**B**) light 14 days, (**C**) light 21 days, (**D**) dark 7 days, (**E**) dark 14 days, (**F**) dark 21 days

GC-MS analysis was performed to understand the effect of light and darkness on the production of pigment-related and antimicrobial compounds in *Streptomyces* sp. VITGV38. Figure 3 shows the peaks of different compounds. The NIST14 library database was utilized to identify the chemical compounds in the pigment extracts obtained under natural light (light and dark) and total darkness (Srinivasan et al., 2017). Seven major pigment-related compounds were detected under both natural light (light/dark) and total dark conditions: indole, benzo(h)quinoline-2,4-dimethyl, n-hexadecanoic acid, tetracosane, pentadecanoic acid, tetradecanoic acid, and 2-piperidinone. The two conditions showed quantitative differences in the abundance of these compounds, with indole, n-hexadecanoic acid, 2-piperidinone, tetracosane, and pentadecanoic acid showing more abundance when the strain was cultured under dark conditions, while benzo(h)quinoline and tetradecanoic acid showed higher abundance when the strain was cultured under natural conditions. As shown in Table 1, a greater number of metabolites were produced under dark conditions at 7 and 21 days, whereas a higher number of compounds were identified under natural light conditions at 14 days.

**Table 1:** Shows the total number of peaks and different pigmented compounds with their retention time and area (%) detected in GC-MS for the two parameters of natural conditions and full dark conditions

S.no	Conditions	Total peaks	Pigment compounds	Retention time (rt)/minutes	Area (%)	Similarity index (SI)
1	Natural 7 days	45 peaks	Indole	12.821	0.65	94%
			Benzo[h]quinoline, 2,4-dimethyl	17.561	1.07	50%
			5-Octadecene, (E)-	17.846	0.34	97%
			7,9-Di-tert-butyl-1-oxaspiro (4,5) Deca-6,9-diene-2,8-dione	19.448	1.32	99%
			.psi., psi.-Carotene, 7,7’,8,8’,11,11’,12,12’,15,15’-decahydro-	25.094	1.26	70%
2	Natural 14 days	45 peaks	Benzo(h) quinoline,2,4-dimethyl	17.561	0.56	50%
			7,9, -Di-tert-butyl-1-oxaspiro (4,5)deca-6,9-diene-2,8-dione	19.448	2.15	99%
			1-Octadecane	18.257	0.49	99%
			Hexadecanoic acid	18.869	6.49	99%
			1-Hexanoyl-pyrrolidine-2-carboxylic acid, diisobutylamide	19.247	0.74	43%
			Piperazine, 1,4-dimethyl	19.582	0.64	47%
			Tetracosane	22.661	0.40	97%
			Pentacosane	22.661	0.47	95%
			Pyrazine, ethyl	24.439	1.19	43%
3	Natural 21 days	43 peaks	2-Piperidinone	11.605	0.56	87%
			Tetradecanoic acid	16.311	0.78	96%
			Pentadecanoic acid	17.393	0.30	96%
			Heptanol	21.721	0.28	38%
			Ethyl 5-chloro-2-nitrobenzoate	22.141	1.38	94%
			Dexrazoxane	28.474	2.33	27%
4	Dark 7 days	45 peaks	Indole	12.813	0.68	95%
			Benzo[h]quinoline, 2,4-dimethyl	18.047	0.65	64%
			n-Hexadecanoic acid	18.945	9.45	99%
			Tetradecanoic acid	16.781	0.34	98%
			3-Octadecane, (E)-	13.870	0.52	91%
			Didecanoic acid	14.532	0.71	99%
			Octadecanoic acid	20.790	2.43	99%
			2-Dodecenol	21.730	0.40	35%
			2,6 diflurobenzoic acid, oct-3-en-2-yl ester	28.164	1.73	30%
5	Dark14 days	45 peaks	Indole	12.813	0.48	95%
			n-Hexadecanoic acid	18.886	6.63	99%
			Tetracosane	21.831	0.48	99%
			Tetradecanoic acid	16.772	0.39	98%
			2-Dodecenol	21.721	0.36	35%
			n-Nonadecanol	16.168	0.36	94%
			Hexacosane	22.661	0.50	94%
			Octadecane	23.458	0.72	97%
			Bis (2-ethylhexyl) phthalate	24.582	2.01	98%
6	Dark 21 days	43 peaks	Indole	12.813	0.56	97%
			n-Hexadecanoic acid	18.425	3.22	89%
			Pentadecanoic acid	16.311	0.36	62%
			Octadecanoic acid	18.869	2.58	93%
			Phenol,o-amino	16.789	0.36	74%
			Tridecanoic acid	18.425	0.56	92%
			Furan, 2-methyl-5(methylthio)	19.062	0.34	50%
			3-pyridine carboxylic acid,1,2,5,6-tetrahydro-1-nitroso-	19.826	1.32	22%

### Antimicrobial activity of the pigment extract

**Figure 4:**
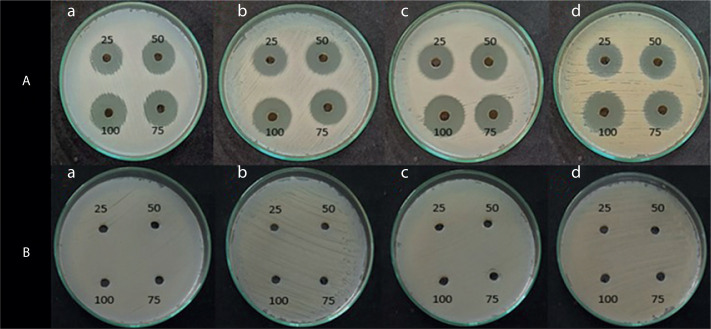
Shows the inhibition zone over the selected microbes against. (**A**) Positive control (tetracycline), and (**B**) negative control (ethyl acetate)

**Figure 5:**
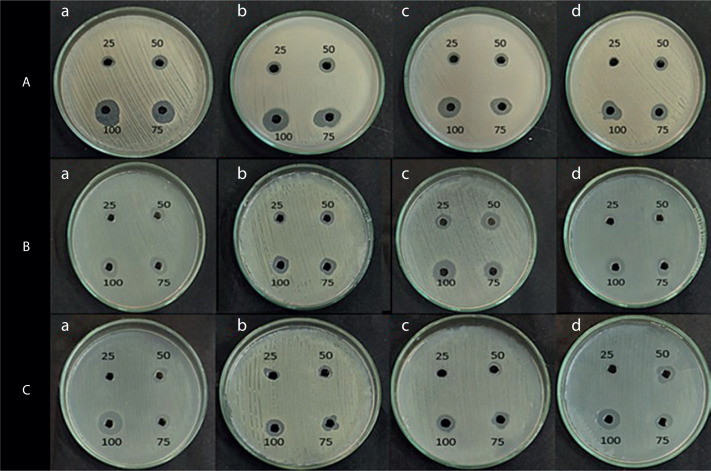
Shows the inhibition zone over the selected microbes against crude extract from VITGV38 natural (light and dark) (**A**) 7 days, (**B**) 14 days, (**C**) 21 days grown in a natural environment

**Figure 6:**
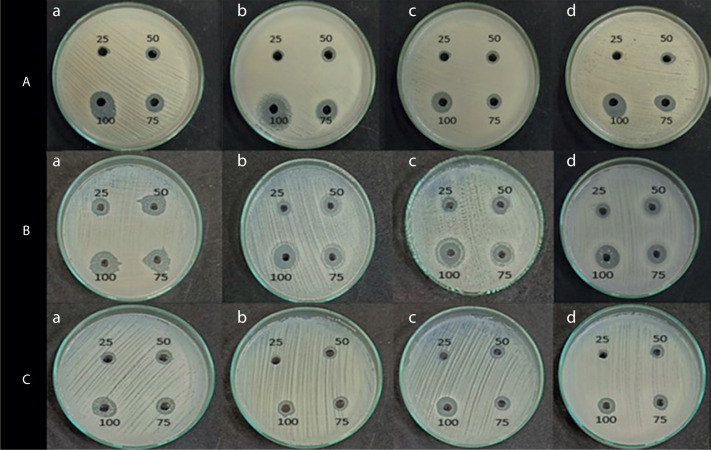
Shows the inhibition zone over the selected microbes against a crude extract from VITGV38 (**A**) 7 days, (**B**) 14 days, (**C**) 21 days grown in a dark environment. (a) Escherichia coli, (b) Staphylococcus aureus, (c) Bacillus subtilis, (d) Pseudomonas aeruginosa

The antimicrobial activity of the pigment extract was tested against four pathogenic bacterial strains: *E. coli, S. aureus, B. subtilis*, and *P. aeruginosa*. The ethyl acetate pigment extracts obtained under natural light (light and dark) and total dark conditions at different time intervals (7, 14, and 21 days) were assessed by the agar well diffusion method. As shown in Figures 4, 5, and 6, the extracts exhibited significant antimicrobial activity, with inhibition zones varying across the light conditions. The positive control (tetracycline) exhibited the highest inhibition zone (22–26 mm at 25–100 µl concentration). The test extracts showed the maximum inhibition (10–20 mm) under the dark condition at 7 days, followed by dark condition (14 days) (inhibition zone: 12–17 mm), natural light condition (7 days) (inhibition zone: 8–17 mm), natural light condition (14 days) (inhibition zone: 11–16 mm), and natural light condition (21 days) (inhibition zone: 9–16 mm). The smallest inhibition zone (8–15 mm) was observed for the pigment extract under the dark condition at 21 days (Table 2). A two-way ANOVA was conducted on the effects of light and dark conditions and varying incubation durations on the antibacterial activity of the pigment extracts. Several extracts, particularly those obtained under dark conditions, showed higher zones of inhibition but without significant differences (*p* > 0.05).

**Table 2:** Zone of inhibition (mm) from VITGV 38 natural conditions (light and dark) and the total dark 7, 14, 21 days of crude extracts for the four selected microbes

Zone of inhibition (mm)																
Sample	*Escherichia coli*				*Staphylococcus aureus*				*Bacillus subtilis*				*Pseudomonas aeruginosa*			
Concentration (µg/ml)	25	50	75	100	25	50	75	100	25	50	75	100	25	50	75	100
Positive control	22	23	23	24	22	23	24	25	22	24	25	26	22	23	24	26
Negative control	–	–	–	–	–	–	–	–	–	–	–	–	–	–	–	–
N 7	8	10	15	17	8	10	13	17	9	10	12	15	–	10	12	15
D 7	-	11	12	15	–	10	12	20	7	10	11	13	–	10	13	17
N 14	8	9	12	13	9	10	11	12	11	13	14	16	8	10	12	13
D 14	12	15	16	17	10	12	13	16	11	14	15	16	10	12	13	17
N 21	9	10	11	16	–	9	10	11	–	9	10	11	8	10	12	13
D 21	8	10	11	13	–	8	10	11	–	11	12	15	–	10	11	12

### Antioxidant activity of the pigment extracts

The DPPH radical scavenging assay was conducted to assess the antioxidant activity of crude pigment extracts at 25, 50, and 100 µg/ml. The radical scavenging activity of the pigment extracts increased in a concentration-dependent manner. Among the tested crude extracts, extract 38 L-21 exhibited the highest percentage of radical scavenging activity (%RSA) at 100 µg/ml (83.66%), followed by extracts 38 L-14 (82.07%) and 38 L-7 (80.79%). The lowest %RSA values were observed for extracts 38 d-7, 38 d-14, and 38 d-21 at 100 µg/ml (range: 77–78%). The standard antioxidant (ascorbic acid) exhibited the highest scavenging efficiency of 97.96% RSA at 100 µg/ml concentration.

**Figure 7:**
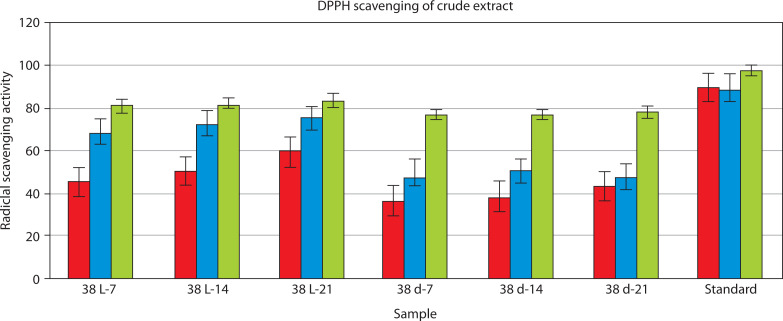
Antioxidant activity of Streptomyces sp. VITGV38 light and dark 7, 14, 21 days and different concentrations

Linear regression equations obtained from the %RSA data were used to calculate the IC_50_ value, which is the concentration required to inhibit 50% DPPH radicals. A strong antioxidant capability is indicated by a lower IC_50_ value. As shown in Figure 7, the 38 L-21 extract with the highest antioxidant activity had the lowest IC_50_ value (0.143 µg/ml) among the evaluated extract. Additionally, two extracts, namely 38 L-7 (1.134 µg/ml) and 38 L-14 (0.854 µg/ml), demonstrated encouraging antioxidant properties. In contrast, the pigment extracts obtained under dark conditions showed greater IC_50_ values: extract 38 d-7 (1.783 µg/ml), extract 38 d-14 (1.713 µg/ml), and extract 38 d-21 (1.615 µg/ml). A one-way ANOVA on the %RSA values at 100 µg/ml concentration revealed that these differences were significant. A highly significant difference between the isolates was found by the study (*F* = 1.46 × 10^29^, *p* < 0.0001), suggesting that strains differed greatly in their antioxidant activity.

### Pharmacokinetic studies

SwissADME analysis was conducted to assess the pharmacokinetic profile, drug-likeness, and toxicity of the nine bioactive compounds derived from *Strepto-myces* sp. VITGV38 by entering their SMILES structures. As shown in Table 3, benzo[h]quinoline-2,4-dimethyl had a molecular weight of 207.27 g/mol, a topological polar surface area (TPSA) of 12.89 Å, and water solubility ranging from soluble to slightly soluble (–4.568 log S). It exhibited high gastrointestinal absorption and considerable blood-brain barrier (BBB) permeability, with a bioavailability score of 0.55%, meeting Lipinski’s rule of five criteria for oral drug-likeness. However, ADMET analysis revealed that while most compounds exhibited moderate to high solubility and permeability, 3-octadecane displayed low solubility (log S = –8.481) and potential bioavailability concerns, as indicated by Ghose and Veber’s filters. Benzo[h]quinoline-2,4-dimethyl was identified as a P-glycoprotein (P-gp) substrate, while most compounds showed favorable permeability across the Caco-2 membrane. BBB permeability prediction indicated that 3-octadecane had a high penetration capacity, whereas other compounds exhibited low to moderate BBB permeability, favoring selectivity in peripheral tissues. Central nervous system (CNS) permeability analysis suggested that these compounds had limited potential for CNS-related applications. Metabolic assessments showed that indole, benzo[h]quinoline-2,4-dimethyl, and octadecanoic acid inhibited CYP1A2 and CYP3A4 enzymes, suggesting a risk of drug-drug interactions. As shown in Table 3, toxicity predictions indicated Ames toxicity for benzo[h]-quinoline-2,4-dimethyl and hepatotoxicity potential for 2,6-difluorobenzoic acid, oct-3-en-2-yl ester. None of the compounds inhibited hERG. According to oral toxicity classification, most compounds were graded as categories IV or V, indicating relatively low acute toxicity in rats. Environmental toxicity assessments suggested reasonable bioaccumulation potential of the compounds. Indole, n-hexadecanoic acid, tetradecanoic acid, dodecanoic acid, and 2-dodecanol exhibited the best safety profiles with good absorption, moderate distribution, metabolic stability, and minimal toxicity risks.

**Table 3:** Physicochemical properties, pharmacokinetics, drug-likeness (SwissADME), and predicted toxicity profiles of nine selected compounds

S. No	Properties	Indole	Benzo[h]quinoline, 2,4-dimethyl	n-Hexa-decanoic acid	Tetrade-canoic acid	3-Octa-decane	Dode-canoic acid	Octade-canoic acid	2-Dode-canol	2,6-Difluorobenzoic acid, oct-3-en-2-yl ester
**Physicochemical properties**										
1	Molecular weight (g/mol)	117.15	207.27	256.42	228.37	254.49	200.32	284.48	186.33	268.30
2	Fraction Csp3	0.00	0.1	0.9	0.9	1.0	0.9	0.9	0.0	0.4
3	No of Rotatable bonds	0	0	14	12	15	10	16	9	7
4	No of H-bond acceptors	0	1	2	2	0	2	2	1	4
5	No of H-bond donors	1	0	1	1	0	1	1	1	0
6	TPSA (Å^2^)	15.7 Å^2^	12.8 Å^2^	37.3 Å^2^	37.3 Å^2^	0.0 Å^2^	37.3 Å^2^	37.3 Å^2^	20.2 Å^2^	26.30 Å^2^
**Lipophilicity**										
7	Log *P*_o/w_ (iLOGP)	1.4	2.6	3.8	3.3	5.2	2.7	4.3	3.5	3.3
8	Log *P*_o/w_ (XLOGP3)	2.0	4.2	7.1	6.1	9.3	4.2	8.2	5.0	4.7
9	Log *P*_o/w_ (MLOGP)	1.5	3.3	4.1	3.6	6.9	3.1	4.6	3.4	4.6
10	Consensus Log *P*_o/w_	1.9	3.7	5.2	4.4	7.1	3.5	5.6	3.9	4.5
**Pharmacokinetics (ADME)**										
**(Absorption)**										
11	Water solubility (log S)	–1.949	–4.568	–5.562	–4.952	–8.481	–4.181	–5.973	–4.769	–4.955
12	Skin permeability (log Kp)	–1.809	–2.306	–2.717	–2.705	–2.644	–2.693	–2.726	–1.495	–2.378
13	P-gp substrate	No	Yes	No	No	No	No	No	No	No
**(Distribution)**										
14	BBB permeability	0.428	0.474	–0.111	–0.027	0.977	0.057	–0.195	0.693	0.435
15	CNS permeability	–1.969	–1.405	–1.816	–1.925	–1.308	–2.034	–1.707	–2.056	–1.794
16	VDss (human)	0.26	0.398	–0.543	–0.578	0.661	–0.631	–0.528	0.371	0.039
**(Metabolism)**										
17	CYP1A2 inhibitor	Yes	Yes	No	No	Yes	No	Yes	No	Yes
18	CYP2C19 inhibitor	No	Yes	No	No	No	No	No	No	Yes
19	CYP2C9 inhibitor	No	Yes	No	No	No	No	No	No	No
20	CYP2D6 inhibitor	No	No	No	No	No	No	No	No	No
21	CYP3A4 inhibitor	No	No	Yes	No	No	No	No	No	No
**(Excretion)**										
22	Total clearance	0.396	0.313	1.763	1.693	1.924	1.623	1.832	1.673	0.411
23	Renal OCT2 substrate	No	No	No	No	No	No	No	No	No
**Drug-likeness**										
24	Lipinski rule	Yes	Yes	Yes	Yes	Yes	Yes	Yes	Yes	Yes
25	Ghose filter	No	Yes	Yes	Yes	No	Yes	No	Yes	Yes
26	Veber filter	Yes	Yes	No	No	No	Yes	No	Yes	Yes
27	Egan filter	Yes	Yes	Yes	Yes	No	Yes	No	Yes	Yes
28	Muegge filter	No	No	No	No	No	Yes	No	No	Yes
29	Bioavailability score	0.55	0.55	0.85	0.85	0.55	0.85	0.85	0.55	0.55
**Toxicity (human, animal, environmental)**										
30	Ames test (mutagenicity)	No	Yes	No	No	No	No	No	No	No
31	hERG I inhibitor	No	No	No	No	No	No	No	No	No
32	hERG II inhibitor	No	No	No	No	No	No	No	No	No
33	Hepatotoxicity	No	No	No	No	No	No	No	No	Yes
34	Max. tolerated dose (log mg/kg/day)	0.575	0.204	–0.708	–0.559	0.066	–0.344	–0.791	0.356	1.437
**Oral rat**										
35	LD50 (oral rat, mg/kg)	1,161	0,806	1,194	1,123	1,373	1,145	1,199	1,428	1,274
36	Oral toxicity classification	IV	IV	V	V	V	V	V	V	IV
**Environmental**										
37	Bioaccumulation factor Log10 (BCF)	0,915	1,932	2,285	1,915	1,938	1,662	2,084	1,703	2,218
38	Daphnia magna LC50-Log10 (mol/l)	4,638	5,048	4,446	4,236	4,937	4,014	4,671	3,509	5,930
39	Fathead minnow LC50 Log10 (mmol/l)	–0,719	–1,864	–3,326	–2,650	–5,310	–1,804	–4,061	–1,904	–3,262
40	Tetrahymena pyriformis IGC50 –Log10 (mol/l)	-0,124	1,438	2,692	2,163	3,723	1,602	3,150	1,260	1,992

### Molecular docking analysis

**Figure 8:**
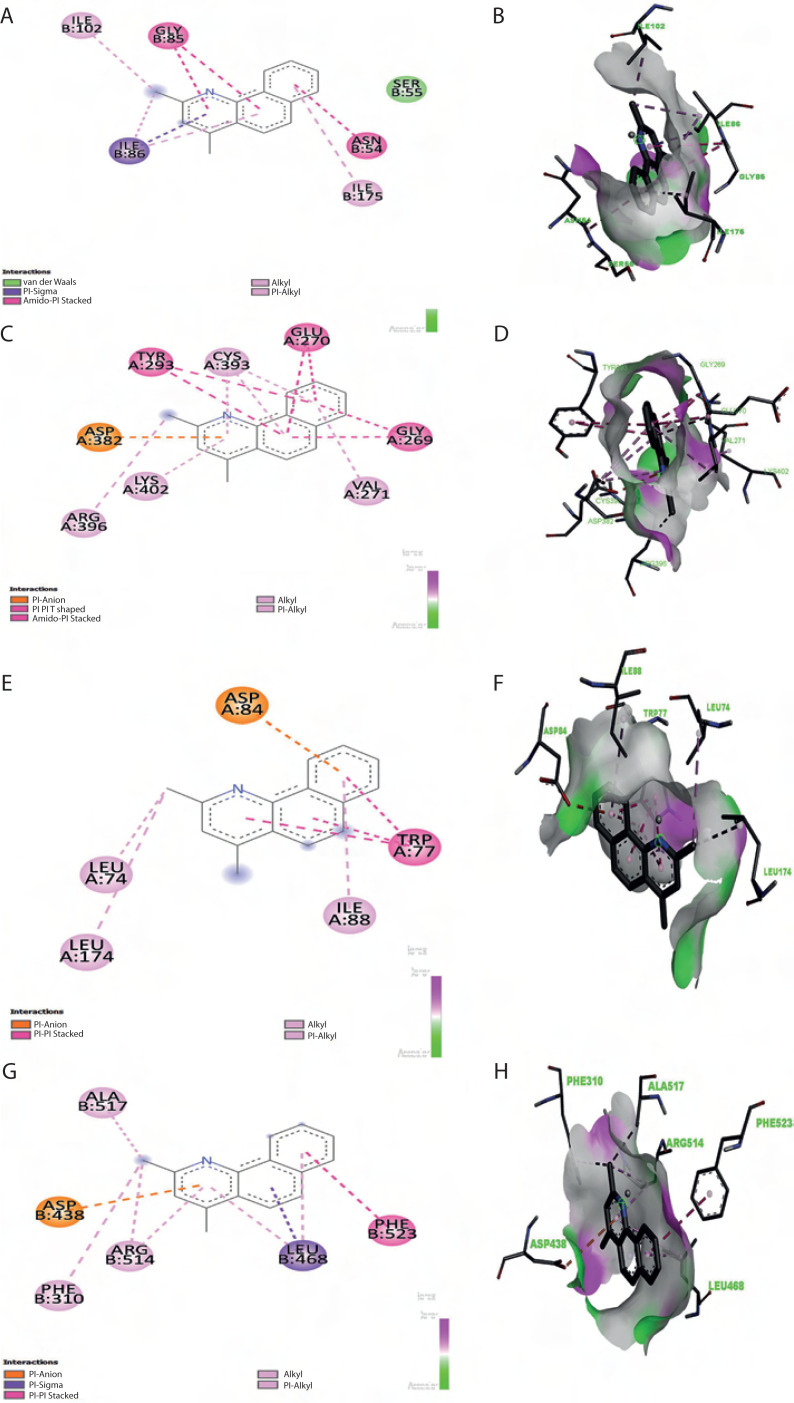
2D and 3D molecular docking analysis of Benzo(h)quinoline-2,4-dimethyl with target proteins. (**A, B**) Enoyl-ACP reductase (3G75) of *Staphylococcus aureus*. (**C, D**) Penicillin-binding protein (3E7X) of *Bacillus subtilis*. (**E, F**) DNA gyrase (5IMJ) of *Escherichia coli*, and (**G, H**) multidrug efflux pump MexB (4QMK) of *Pseudomonas aeruginosa*. 2D interaction diagrams illustrate key binding interactions, while 3D docking poses show ligand orientation within the active site

To evaluate target protein binding, it is crucial to assess macromolecule-ligand interactions by using computational approaches before developing new drugs. Molecular docking, performed using PyRx and AutoDock Vina, enables us to identify reliable docked poses and binding scores between receptors and ligands. Because AutoDock Vina is widely used in docking applications, the consistency of interpretations further supports the accuracy of the results. Here, nine primary bioactive compounds identified through mass spectrometry were docked with selected bacterial target proteins using AutoDock methods. As summarized in Table 4, the docking analysis revealed that benzo(h)quinoline-2,4-dimethyl, 2,6-difluorobenzoic acid, and indole exhibited strong binding affinities, stable molecular interactions, and remarkable hydrogen bonding with protein-binding residues. The docked protein-ligand combination of the pigment extract showed a minimum binding energy of –8.4 kcal/mol in *B. subtilis*, exceeding that of tetracycline (–7.7 kcal/mol), while 2,6-difluorobenzoic acid exhibited competitive binding scores. Key molecular interactions revealed that crucial binding residues contribute to the antibacterial activity of these compounds. As illustrated in Figure 8, benzo(h)quinoline-2,4-dimethyl interacted strongly with critical residues in *E. coli* (ASP 84, LEU 74, ILE 88, and TRP 77), *S. aureus* (GLY 85, ILE 102, and ASN 54), *B. subtilis* (GLU 270, ASP 382, and ARG 396), and *P. aeruginosa* (ALA 517, ARG 514, and PHE 310). Similarly, 2,6-difluorobenzoic acid exhibited stable interactions with key residues in *E. coli* (PHE 111, ARG 116, and ASP 230), *S. aureus* (LEU 103, ILE 51, and ASP 81), and *P. aeruginosa* (LYS 633, ALA 634, and PHE 624). Tetracycline, used as a reference antibiotic, displayed strong interactions with known bacterial target residues. The redocking of the original ligands into their respective protein binding sites yielded RMSD values below 2.0 Å, indicating the successful reproduction of the experimentally observed poses. Specifically, the RMSD values for 5IMJ, 4QMK, 3G75, and 3E7X were 1.119 Å, 1.967 Å, 1.761 Å, and 1.627 Å, respectively. These values indicate a high degree of alignment between the redocked and crystal poses, confirming that the molecular docking protocol used in this study was reliable and precise. Fatty acids such as n-hexadecanoic acid, dodecanoic acid, and octadecanoic acid exhibited moderate binding affinities, indicating a potential secondary antibacterial role, possibly through membrane disruption. In contrast, 3-octadecane showed weak binding affinity, suggesting limited antimicrobial potential. As shown in Table 4, the results highlight benzo(h)quinoline-2,4-dimethyl and 2,6-difluorobenzoic acid as promising lead compounds for antimicrobial drug development.

**Table 4:** Molecular docking binding affinity and interaction

Proteins	Parameters	Indole	Benzo(h) quinoline 2,4 dimethyl	n-Hexade-canoic acid	Tetra-decanic acid	3 octadecane	Dodecanoic acid	Octadecanoic acid	2 dode-canol	2,6-diffluro benzoic acid	Tetra-cycline
5IMJ (Escherichia coli)	Binding affinity	–5	–7	–4.2	–4.8	–3.9	–4.4	–4.4	–4.1	–5.8	–7.4
	Interactions	GLU 22, LEU 172, ILE 25, LEU 168	ASP 84, LEU 74, LEU 174, ILE 88, TRP 77	LEU 125	LEU 117, LEU 229, TRP 142, LEU 145, ARG 116	HIS 37, PHE 23, CYS 132, LEU 19, HIS 140	ASP 36, GLN 27, ALA 38, HIS 37, PHE 23, LEU 19, CYS 132	VAL 35, PRO 33	PRO 128, ARG 45, ARG 45, LEU 125, GLU 49, PRO 128	PHE 111, ARG 116, ASP 230, LEU 117, LEU 229	ARG16 ARG56 VAL53 PRO126 ILE127 GLU13
3G75 (Staphylococcus aureus)	Binding affinity	–5.1	–7.6	–5.2	–5.2	–4.3	–4.7	–4.7	–4.8	–6.7	–6.4
	Interactions	ASP 81, SER 55, ILE 175	GLY 85, ILE 102, ILE 86, SER 35, ASN 54, ILE 175	SER 129, LEU 103, ILF 175, ILE 102, ASN 54, THR 173, ILE 86, ILE 51, ASP 81, GLU 58, PRO 87, GLY 85, ARG 84, GLY 83, SER 55	THR 173, ILE 102, LEU 103, ILE 86, ILE 51, ILE 175, GLY 85, ARG 84	ILE 175, ILE 86, LEU 103, ILE 51	ASN 54, ILE 51, ILE 175, ILE 86	LEU 103, ILE 102, SER 55, ASP 81, ILE 86	LEU 103, ILE 175, ILE 86, ILE 51, ARG 84	LEU 103, ILE 51, VAL 79, ILE 175, ASP 81, THR 173, GLY 85, ILE 86	THR104 LEU25 GLY24
3E7X (Bacillus subtilis)	Binding affinity	–5.9	–8.4	–5	–5	–4.2	–5	–4.9	–4.7	–5.8	–7.7
	Interactions	TYR 406, ALA 191, THR 241, HIS 404	GLU 270, CYS 393, TYR 293, GLY 269, ASP 382, ARG 396, LYS 402, VAL 27	ARG 396, GLN 400, LYS 402, VAL 271	ASP 382, ARG 396, LYS 402, VAL 271, VAL 437, ILE 401	PHE 179, PHE 290, VAL 305, VAL 311, PHE 322	ASN 291, GLU 270, TYR 293, CYS 393, VAL 271, LYS 402	HIS 404, PRO 192, LYS 217, VAL 220	ARG 396, LYS 159, LYS 367, LEU 364, ALA 368	PRO 295, TYR 293, CYS 393, ARG 396, LYS 402	TYR358 TYR378 LYS159 ARG379 ASP398
4QMK (Pseudomonas aeruginosa)	Binding affinity	–5.3	–7.4	–4.5	–4.5	–4.3	–4.6	–4.4	–4.6	–5.8	–7.4
	Interactions	GLU 422, SER 50, VAL 49, PRO 51	ALA 517, ASP 438, ARG 514, PHE 310, LEU 468, PHE 523	GLU 630, ALA 131, PHE 624	TRP 510, LEU 468, ARG 514, ALA 517, PRO 518, LYS 513	PHE 523, LEU 468, ARG 514, PHE 310, ALA 517	LEU 35, MET 440, ARG 33, LYS 317	LYS 513, ALA 435, PHE 426, PRO 314	GLN 469, TRP 510, ALA 517, ARG 514, LEU 468	LYS 633, ALA 634, SER 146, GLU 630, PHE 624	ARG575 ARG616 SER625 TRP620 LYS618 GLY241

## Discussion

Pigments are an important source for preparing colors. The dye industry seeks novel pigment-producing organisms. Actinomycetes are a rich source of bioactive compounds and pigments (Parmar et al. 2017). In the present study, *Streptomyces* sp. VITGV38, isolated from a tomato plant, was found to produce red to dark brown pigments. It formed circular colonies, with a powdery appearance, and produced smooth grey spores. SEM results revealed compartmentalized hyphae with glossy spore surface, and the spores were chain-like and arranged linearly. Each chain contained around 10 to 20 spores in the spiral-shaped style, similar to that observed in *Streptomyces* sp. MCCB 267 (Dhaneesha et al. 2019). This study extends previous research on the antibacterial potential of secondary metabolites produced by *Streptomyces* sp. VITGV38 (MCC 4869) (Hussain and Christopher 2023). Here, we investigated the capacity of this strain to produce pigments under various light conditions as well as the antibacterial and antioxidant properties of the pigment-related compounds. In contrast to the previous study, we used GC-MS to identify bioactive pigment metabolites and assessed their drug-likeness by conducting ADME/toxicity analysis and molecular docking analysis. The results demonstrated that *Streptomyces* sp. VITGV38 is a valuable source of natural pigments and antibacterial compounds, with potential uses in both industry and medicine. *Streptomyces* sp. VITGV38 was cultured in natural light (light/dark) and total dark conditions to examine the effects of these physical elicitors on pigment production. The optimal pH of 7.4 was maintained during the culturing process. Light conditions enhance the development and synthesis of pigments. Some strains are light-sensitive and might exhibit cell damage following visible light exposure. Regarding this aspect, we found no evidence of the harmful effect of light on filamentous germination bacteria such as *Streptomyces*. As shown previously, mycelium and spore formation are also influenced by light. This finding suggests that light is an important environmental signal for microorganisms (Imbert and Blondeau 1999).

We also conducted GC-MS analysis to reveal the chemical composition of the obtained pigment extracts and identify the potential and chemical compounds involved in their biological activities. Compared to culturing under natural light (light/dark), culturing under total darkness resulted in the production of a larger variety of pigment-related compounds. As reported previously, dark colors such as yellow, benzo[h]quinoline-2,4-dimethyl, and 7,9-di-tert-butyl-1-oxaspiro(4,5) deca-6,9-diene-2,8-dione are produced by *S. filamentous* strain KS17 (Chakraborty et al. 2022); psi.,.psi.-carotene, 7,7’,8,8’,11,11’,12,12’,15,15’-decahydro-, are produced by *Streptomyces* sp. 1S1 (Kamel et al., 2023); n-hexadecanoic acid is produced by *Streptomyces anulatus* (El-Naggar et al. 2017a); brown indole with red to brown color is produced by *Streptomyces* sp. VITGV100 (Veilumuthu et al. 2022); 5-octadecene, (E)-, a compound with potential antimicrobial properties, is produced by *S. cheonanensis*(Ferdosi et al. 2022); and 1-octadecene is produced by *S. rochei* SUN35 (Awad et al. 2023) Ethyl 5-chloro-2-nitrobenzoate is produced under natural conditions. Moreover, as reported previously, under dark conditions, colors such as white and n-nonadecanol-1 were produced by *Streptomyces* sp. HB084 (Venugopal et al. n.d.), n-hexadecanoic acid is produced by *Streptomyces anulatus* (El-Naggar et al. 2017b), and yellow (octadecanoic acid) is also produced by *S. anulatus* NEAE-94 (El-Naggar et al. 2017c). Bis(2-ethylhexyl) phthalate is produced by *S. bangladeshensis*, which acts as an antimicrobial agent (Al-Bari et al. 2005). 3-Pyridine carboxylic acid from *Nocardia species* 236 has also been reported previously (Lu et al. 2011). High production of light brown (2,6-difluorobenzoic acid, oct-3-en-2-yl ester and 3-octadecene, (E)-) and several compounds produced under natural and dark conditions have been reported in previous studies.

In the present study, the compound that appeared at retention time –17.561 (on the 7^th^ day and 14^th^ day of natural light condition) and –18.047 (on the 7^th^ day of dark condition) exhibited a low similarity index to benzo(h)quinoline-2,4 dimethyl in the GC-MS analysis, indicating that it did not strongly match with any known compounds in the database. However, it is closely associated with the benzoquinoline group, which is known for various biological activities. This compound was selected for further studies as it was one of the pigmented compounds produced by *Streptomyces* sp. VITGV38, indicating its potential bioactivity.

The crude pigment extract obtained on the 7^th^ day under dark conditions showed an inhibition zone of 20 mm, which is consistent with previous studies reporting similar inhibition zones for extracts (de Azevedo et al. 2024). This finding suggests that the pigment extracts contained bioactive compounds with potential antimicrobial properties. Previous studies have reported IC_50_values for *Streptomyces* extracts (Rammali et al. 2024; Singh et al. 2022; Tan et al. 2015); the present study also found significantly lower IC_50_ values for several extracts, highlighting their superior antioxidant potential.

SwissADME analysis was conducted using SMILES input for the nine bioactive compounds from *Streptomyces* sp. VITGV38. Table 3 provides comprehensive insights into the pharmacokinetic profiles, drug-likeness, and toxicity of these compounds. This in silico ADME/toxicity screening approach, which utilizes the established Lipinski rule of 5 criteria, is fundamental for assessing a compound’s potential as a lead candidate based on the evaluation of critical parameters such as molecular weight, hydrogen bonding, and lipophilicity that regulate drug absorption and distribution (Lipinski et al. 1997). In the present study, all compounds met Lipinski’s criteria for oral bioavailability, although 3-octadecane and octadecanoic acid exhibited potential bioavailability issues based on Ghose and Veber’s filters, with 3-octadecane showing particularly low solubility (log S = –8.481). Notably, benzo[h]quinoline-2,4-dimethyl (MW: 207.27 g/mol; TPSA: 12.89 Å; log S = –4.568) exhibited high gastrointestinal absorption, substantial BBB permeability, and a bioavailability score of 0.55% (Ngbolua et al. 2022), highlighting its potential as an oral therapeutic agent despite its status as a P-glycoprotein substrate, which may predispose it to efflux-mediated resistance. Metabolic predictions indicated that indole, benzo[h]quinoline-2,4-dimethyl, and octadecanoic acid inhibited CYP1A2 and CYP3A4 enzymes, suggesting a potential for drug-drug interactions, while toxicity assessments revealed Ames toxicity for benzo[h]quinoline-2,4-dimethyl and hepatotoxicity potential for 2,6-difluorobenzoic acid derivatives, although none of these compounds inhibited hERG channels and most of them were grouped into low acute toxicity categories. Moreover, based on established guidelines, quantitative predictions of ADME/toxicity characteristics, including a reported minimum binding score of –5.8 against cyclin-dependent kinase-2 (CDK2, PDB ID: 2R3J) (Yadav et al. 2016; Saurav et al. 2014), further support the therapeutic potential of these compounds. Collectively, these findings highlight the promising pharmacokinetic profiles of the isolated compounds while identifying key areas, namely solubility, metabolic stability, and toxicity reduction, which require further optimization to enhance their development as effective therapeutic agents.

Molecular docking, a critical technique in drug discovery, allows to predict the strength of ligand-protein interactions (Olaokun and Zubair 2023). Potential drug candidates are rapidly identified through virtual screening and docking, which simulate their binding to target proteins by using tools such as AutoDock. These methods facilitate the identification of potential molecules for additional research, thereby expediting the drug discovery process (Naithani and Guleria 2024). In the present study, molecular docking analysis was conducted for the nine bioactive compounds from *Streptomyces* sp. VITGV38 by using PyRx and AutoDock Vina against bacterial proteins DNA gyrase (5IMJ) of *E. coli*, enoyl-ACP reductase (3G75) of *S. aureus*, penicillin-binding protein (3E7X) of *B. subtilis*, and the multidrug efflux pump MexB (4QMK) of *P. aeruginosa*, and the findings revealed promising antibacterial potential of these compounds. These targets, selected for their pivotal roles in DNA replication, fatty acid synthesis, cell wall biosynthesis, and drug efflux, respectively, provide a robust framework for evaluating the efficacy of antimicrobial agents. Notably, benzo[h]quinoline-2,4-dimethyl exhibited the highest binding affinity, with a binding energy of –8.4 kcal/mol against *B. subtilis*, compared to –7.7 kcal/mol binding energy of tetracycline. 2,6-Difluorobenzoic acid and indole also showed competitive binding and stable interactions characterized by remarkable hydrogen bonding. The key amino acid residues involved in the interactions were ASP, LEU, ILE, and TRP in *E. coli*; GLY, ILE, and ASN in *S. aureus*; and GLU, ASP, and ARG in *B. subtilis*; this finding suggests the formation of stable inhibitory complexes likely facilitated by structural features such as methyl and hydroxyl groups (Adem Endris et al. 2024). To validate the reliability of the docking method used in this study, we performed redocking of the original co-crystallized ligands. The RMSD values between the redocked and native ligand poses were calculated to evaluate the accuracy of the docking protocol. The obtained RMSD values [1.119 Å (5IMJ), 1.967 Å (4QMK), 1.761 Å (3G75), and 1.627 Å (3E7X)] were below the commonly accepted threshold of 2.0 Å. This finding indicates that the docking approach successfully reproduced the experimentally determined binding poses and supports the use of AutoDock Vina with PyRx for virtual screening. As reported previously, RMSD values of < 2.0 Å reflect a valid and reliable docking performance (Hevener et al. 2009; Plewczynski et al. 2011). These findings confirmed that the docking results of *Streptomyces*-derived secondary metabolites with the target bacterial proteins are valid for further interpretation and drug development analysis. The docking protocol was validated by redocking the native ligands into their respective protein binding sites, resulting in RMSD values below the standard threshold of 2.0 Å. Specifically, the RMSD values for 5IMJ, 4QMK, 3G75, and 3E7X were 1.119 Å, 1.967 Å, 1.761 Å, and 1.627 Å, respectively, reflecting strong concordance with the crystallographic binding poses. This level of accuracy aligns with the criteria established by Hevener et al. (n.d.), who report that RMSD values under 2.0 Å indicate reliable and precise docking outcomes. These results confirm that the molecular docking approach employed, using AutoDock Vina via PyRx, is suitable and dependable for modeling ligand-protein interactions. Thus, the docking predictions for the *Streptomyces* strain-derived secondary metabolites are reliable and support their potential as bioactive candidates for further experimental validation. Overall, these *in silico* findings highlight the promising potential of these natural bioactive molecules as lead candidates for antimicrobial drug development, although further *in vitro* and *in vivo* validations, together with detailed structure-activity relationship studies, are essential to confirm their efficacy and address drug resistance challenges (Trott and Olson 2010; Morris et al. 2009).

## Conclusions

This study identifies *Streptomyces* sp. VITGV38 as a possible source of bioactive pigments, specifically benzo[h]quinoline-related compounds with robust antibacterial properties. These compounds have the promising potential for further transformation into a safe and nontoxic antimicrobial drug for large-scale commercial production. Molecular docking analysis validated that this compound interacts with bacterial target proteins; moreover, natural light conditions increase pigment production. Further purification and safety validation are required to support its potential use in industrial applications and drug development.
